# Performance of Microscopy for the Diagnosis of Malaria and Human African Trypanosomiasis by Diagnostic Laboratories in the Democratic Republic of the Congo: Results of a Nation-Wide External Quality Assessment

**DOI:** 10.1371/journal.pone.0146450

**Published:** 2016-01-20

**Authors:** Pierre Mukadi, Veerle Lejon, Barbara Barbé, Philippe Gillet, Christophe Nyembo, Albert Lukuka, Joris Likwela, Crispin Lumbala, Justin Mbaruku, Wim Vander Veken, Dieudonné Mumba, Pascal Lutumba, Jean-Jacques Muyembe, Jan Jacobs

**Affiliations:** 1 National Institute of Biomedical Research, Kinshasa, Democratic Republic of the Congo; 2 University of Lubumbashi, Lubumbashi, Democratic Republic of the Congo; 3 Institut de Recherche pour le Développement, Montpellier, France; 4 Department of Clinical Sciences, Institute of Tropical Medicine, Antwerp, Belgium; 5 National Malaria Control Program, Kinshasa, Democratic Republic of the Congo; 6 National Human African Trypanosomiasis Control Program, Kinshasa, Democratic Republic of the Congo; 7 University of Kinshasa, Kinshasa, Democratic Republic of the Congo; 8 Department of Microbiology and Immunology, KU Leuven, Leuven, Belgium; Royal Tropical Institute, NETHERLANDS

## Abstract

The present External Quality Assessment (EQA) assessed microscopy of blood parasites among diagnostic laboratories in the Democratic Republic of the Congo. The EQA addressed 445 participants in 10/11 provinces (October 2013–April 2014). Participants were sent a panel of five slides and asked to return a routinely stained slide which was assessed for quality of preparation and staining. Response rate was 89.9% (400/445). For slide 1 (no parasites), 30.6% participants reported malaria, mostly *Plasmodium falciparum*. Only 11.0% participants reported slide 2 (*Plasmodium malariae*) correctly, 71.0% reported “malaria” or “*Plasmodium falciparum*” (considered acceptable). Slide 3 contained *Plasmodium falciparum* (109/μl) and *Trypanosoma brucei brucei* trypomastigotes: they were each reported by 32.5% and 16.5% participants respectively, 6.0% reported both. Slide 4 (*Trypanosoma*) was recognised by 44.9% participants. Slide 5 (*Plasmodium ovale*) was correctly reported by 6.2% participants, another 68.8% replied “malaria” or “*Plasmodium falciparum*” (considered acceptable). Only 13.6% of routine slides returned were correctly prepared and stained. The proportion of correct/acceptable scores for at least 4/5 slides was higher among EQA-experienced participants compared to first time participants (40.9% versus 22.4%, *p* = 0.001) and higher among those being trained < 2 years ago compared to those who were not (42.9% versus 26.3%, *p* = 0.01). Among diagnostic laboratories in Democratic Republic of the Congo, performance of blood parasite microscopy including non-*falciparum* species and *Trypanosoma* was poor. Recent training and previous EQA participation were associated with a better performance.

## Introduction

The detection of *Plasmodium* parasites by light microscopy is still the primary method of malaria diagnosis in most health care facilities throughout the world [1]. Giemsa-stained thick blood film analysis is cheap and enables to score parasite density, to identify the different *Plasmodium* species and to differentiate sexual (gametocytes) from asexual stages. Several other blood parasites can be also detected by microscopic examination of stained blood films, including *Trypanosoma brucei* spp. causing human African trypanosomiasis (HAT). However, microscopy is labour-intensive and its quality in endemic settings is often inadequate due to poor-quality stains and equipment (including but not limited to microscopes), poor maintenance, insufficient training (shortage of competent staff) or a lack of quality assurance [2–5].

With 207 million of episodes of malaria reported in 2012 [6], the Democratic Republic of the Congo (DRC) is a central African country with one of the highest malaria burdens in the world [7]. About 80% of all reported HAT cases in the world occur in the DRC [8]. Whereas prior to 2008, screening for HAT was mainly done by specialized mobile teams of National Control Program for HAT (Programme National de Lutte contre la Trypanosomiase Humaine Africaine, PNLTHA), HAT diagnosis–comprising serological testing followed by parasite detection- is now being integrated into the country’s health system [9,10].

Quality control programs are a prerequisite of competent microscopy. Apart from cross-checking of blood films recommended by World Health Organization (WHO), External Quality Assessment (EQA) is a powerful tool to monitor quality of laboratory diagnosis [1]. EQA sessions provide an overview of the performance levels of end-users and assess the type of common errors allowing targeted training and design of job aids. In addition, EQA provides a valuable educational stimulus and may give insights in the general performance levels of laboratories and, in case, diagnostic kits [11].

Since 2010, two nationwide EQA sessions addressing Giemsa stained blood microscopy have been organized in DRC [12,13]; they revealed the poor quality of malaria microscopy. After refreshment training of diagnostic laboratories, a third nationwide EQA of Giemsa stained blood films microscopy was organized, of which the results are presented in this paper.

The objectives of the EQA were (i) to assess correct reading and interpretation of Giemsa stained thick and thin blood films for the diagnosis of malaria as well as HAT among diagnostic laboratories, (ii) to compare performances of laboratories which participated to the previous and present EQA, and (iii) to assess the quality of sample preparation and staining. The objective of the questionnaire was to generate additional information related to diagnostic microscopy such as quality and procurement of stains and reagents.

## Methods

### Design

The EQA was performed from October 2013 till April 2014 in 10 of 11 provinces in DRC; the province of Nord-Kivu was not included because of security reasons. The materials comprised (i) a panel of Giemsa stained thick and thin blood film slides and (ii) a questionnaire addressing practices of diagnostic microscopy.

### EQA samples (stained thick and thin blood films)

The EQA panel consisted of five slides, prepared from left-overs of routinely drawn EDTA-blood samples obtained from patients presenting at INRB or Institute of Tropical Medicine of Antwerp (ITM) as previously described [12]. One *P*. *falciparum* patient sample was spiked with laboratory-cultured *Trypanosoma brucei brucei*. A second blood film containing trypanosomes was prepared from rodent’s blood infected with *Trypanosoma brucei brucei* and diluted in human EDTA blood.

Thin and thick blood films of each sample were applied on a single slide (Menzel- Gläzer Braunschweig, Germany). Fixation of thin blood films was done with methanol (Panreac, Barcelona- Spain), and thin and thick blood films were stained with Giemsa pH 7.2 (Merck, Darmstadt- Germany). Parasite densities were assessed by two expert microscopist on 10 slides and mean ± 2SD counts were calculated [1,14]. Diagnosis of malaria and *Plasmodium* species identification were confirmed by *Plasmodium*-specific polymerase chain reaction (PCR) [15]. The blood films were covered with Entellan (Merck, Darmstadt, Germany) and a cover slip and slides were packed in plastic boxes and distributed within 90 days after preparation.

The reference results for the samples are displayed in Table 1 and were as follows: slide 1: no parasites; slide 2: *Plasmodium malariae*; slide 3: *Plasmodium falciparum* (trophozoites, 109 ± 107/μl) combined with *Trypanosoma* spp. (trypomastigotes); slide 4: *Trypanosoma* spp. (trypomastigotes); slide 5: *Plasmodium ovale*. Parasite densities for slide 2 (*P*. *malariae*) and slide 5 (*P*. *ovale*) were 6,298 ± 1,162 per μl and 1, 938 ± 326 per μl respectively, they contained trophozoites and schizonts as well as–in the case of *P*. *malariae*—gametocytes. For slides 3 and 4, the densities of trypomastigotes on the thick blood film were 1 to 3 parasites per high-power microscopic field and 0–1 parasite per 4 microscopic fields respectively.

**Table 1:** Composition of the external quality assessment and results for each slide.

Slide number. Reference results, Numbers of eligible answers	Numbers of participants categories of correct, acceptable or incorrect answers		
	Correct	Acceptable	Incorrect
1. No parasite observed, n = 392	269 (68.6%)	-	73 *P*. *falciparum*, 47 *Plasmodium* or “positive”
			3 Others (microfilaria and *Trypanosoma*)
2. *Plasmodium malariae*, (6,298 ± 1,162/μl) n = 383	42 (11.0%)	187 *P*. *falciparum* or mixed infection	69 No parasites observed
		10 *P*. *ovale* or *P*. *vivax*	
		75 *Plasmodium* or “positive” (1 with microfilaria)	
*3*. *Plasmodium falciparum*, (109 ± 107/μl) and *Trypanosoma*, (1–3/microscopic field) n = 382	1 (0.3%)	15 *Trypanosoma* and *P*. falciparum*	107 *P*. *falciparum* (including 5 *P*. *falciparum* and *P*. *malariae*)
		7 *Trypanosoma* and Plasmodium*	83 *Plasmodium* or “positive” (including 18 *P*. non-*falciparum*)
		1 *Trypanosoma* and *P*. *falciparum* and *P*. malariae*	36 *Trypanosoma* (including 1 with microfilaria)
			3 *Trypanosoma* and *Plasmodium* non- *falciparum*
			5 Others (microfilaria)
			124 No parasites observed
4. *Trypanosoma*, (0–1/4 microscopic fields) n = 394	171 (43.4%)	6 *Trypanosoma* & *Plasmodium* spp.	40 *P*. *falciparum* (included 1 with microfilaria)
			39 *Plasmodium* (including 1 with microfilaria) or “positive”
			5 Others (microfilaria)
			133 No parasites observed
5. *Plasmodium ovale*, (1,938 ± 326/μl) n = 388	24 (6.2%)	155 *P*. *falciparum* and mixed infection	2 Others (1 *Trypanosoma*, 1 microfilaria)
		41 *P*. *malariae* and *P*. *vivax*	95 No parasites observed
		69 *Plasmodium* or “positive”	
		2 *P*. *falciparum* and microfilaria	

*: The *P*. *falciparum* densities were > or < 2 SD (n = 14) or no parasite densities were reported (n = 9)

Participants were asked to report the diagnosis, in the case of malaria the *Plasmodium* species identification and–in the case of *Plasmodium falciparum—*the parasite density expressed as number of asexual parasites per microliter of blood. In addition, participants were asked to select a routinely processed thick blood film of their own laboratory (the first positive *Plasmodium falciparum* slide collected during the day of survey) and to return it to INRB. This slide was used to assess the quality of preparation and staining: slides were assessed by two microscopists and scored according to six criteria: the dimension and thickness of the film, the intactness of thick blood film, haemolysis of the red blood cells, the staining and distribution of white blood cells and the presence of Giemsa stain precipitates) [1,12,16]. Discordant results were assessed by a third observer and the consensus result was considered.

### Questionnaire

The EQA questionnaire (S1 File) addressed the following topics: (i) the places of microscopy and rapid diagnostic test (RDT) in the diagnosis of malaria and HAT, (ii) if and when training had been received on malaria and HAT diagnosis (iii) participation in the previous EQAs conducted in 2010 and 2011 respectively, further referred to as EQA2010 and EQA2011 respectively [12,13], (iv) the numbers of test performed in the first half of 2013 and their positivity rate, (v) brand/product of stains and their supply and (vi) buffer and its preparation procedure. Answers could be formulated either as closed (answers in multiple choice question (MCQ) format) or open answers.

### Participating health facilities

Proficiency testing of diagnostic laboratories is not yet formalized in DRC. Being the reference laboratory for the national malaria control program (PNLP), the INRB organizes since 2010 EQA sessions to which diagnostic laboratories subscribe on a voluntary and case-by-case basis, mainly through personal professional contacts with PLNP and INRB staff as well through the National Tuberculosis Control Program (Programme Nationale de la Lutte contre la Tuberculose, PNLT) [13,17]. Compared to previous EQA sessions, additional laboratories were recruited based on voluntary participation, accessibility and travel opportunities (for instance, during supervision visits or trainings).Based on the available budget and the availability of collaborators on-site for diffusion, a total of 450 diagnostic laboratories were targeted.

### Shipments

Slide boxes, questionnaire, procedure of parasite density counting, instructions and informed consent form were packed in protected envelopes (Air Pro 4, Propac, Malmo, Sweden) according to UN 3373 standards and shipped by private air carrier to the provincial airports where they were received by the provincial collaborators, who were staff members from the provincial sections of PNLP and PNLT or representatives of the Provincial Division of the Ministry of Health. For Kinshasa and eastern part of Bas-Congo province, envelopes were delivered by staff on-site after travel by car or motorcycle. The provincial collaborators visited the laboratories and had a meeting with the laboratory supervisor to explain objectives and procedure of the EQA and for asking consent. The participating laboratories read the EQA slides, reported the result and answered the questionnaire during the days following the visit of co-investigator. The results and questionnaire forms, together with the positive thick blood film, were packed in the same envelopes, picked up by the provincial collaborators and shipped to the study coordinator.

### Data entry and analysis

The results of the EQA samples, questionnaire answers and thick blood film quality assessment were entered in an Excel spread sheet (Microsoft Corporation, Redmond, Washington, USA). Results for the microscopic diagnosis (slides 1, 2, 3, 4 and 5) were categorized according to the potential impact on patient management and taking into account the predominance of *P*. *falciparum* in DRC (holo-endemic, with *P*. *ovale* and *P*. *malariae* accounting for < 5% of cases [18]).

The following categories were used: correct, acceptable and incorrect.”Correct” meant fully complying with the reference result (diagnosis of malaria and HAT, correct *Plasmodium* species, parasite density within 2 SD of the reference value); “Acceptable” included the correct diagnosis of malaria or *Plasmodium* (but irrespective of correct *Plasmodium* species and density, with the exception of misdiagnosing *P*. *falciparum* as non-*falciparum*) as well as the diagnosis of HAT. For instance, replying “*P*. *vivax*” for slide 5 (expected result: *P*. *ovale*) was considered as “acceptable”. In addition, some participants answered “positive”, *i*.*e*. meaning “positive for malaria”, without species identification–they were grouped together with “*Plasmodium* species”. Finally, there was the category “incorrect” which grouped all other answers; for instance “negative” (*i*.*e*. no parasites observed) thus missing diagnosis of malaria or HAT for slides 2–5, as well as reporting blood parasites in the case of slide 1 or reporting *P*. *falciparum* as non-*falciparum* in slide 3.

Differences between proportions were tested for significance using the Pearson’s Chi-square test using STATA 10.0 (Statacorp, Texas, USA). A *p*-value < 0.05 was considered significant. In addition, results were compared between provinces and according to health facility hierarchy.

Provinces considered HAT endemic included Kinshasa, Bas-Congo, Bandundu, Province Orientale, Equateur, Kasaï oriental and Kasaï Occidental, while Katanga, Sud-Kivu and Maniema were not considered endemic for the disease [13].

### Ethics Statement

The study protocol was approved by the Institutional Review Board (IRB) of the Institute of Tropical Medicine (IRB/AB/ac/096, Ref: 890/13 of 5/6/2013). The participation was voluntary and there was implicit consent for participating to the EQA and questionnaire. The identity of the participants was encoded, with the code only known by the study coordinator. For the consent of the participants, the system of “presumed consent” was used and patient’s samples and data were de-identified. For the *in vivo* culture of trypanosomes in rodents, ethical approval was obtained from the veterinary ethics committee of the Institute of Tropical Medicine Antwerp under protocol BM2013-7.

## Results

### EQA participating health facilities and cost

The present EQA was sent out on October 22^nd^ 2013 and was closed after 21 weeks.

**Fig 1:**
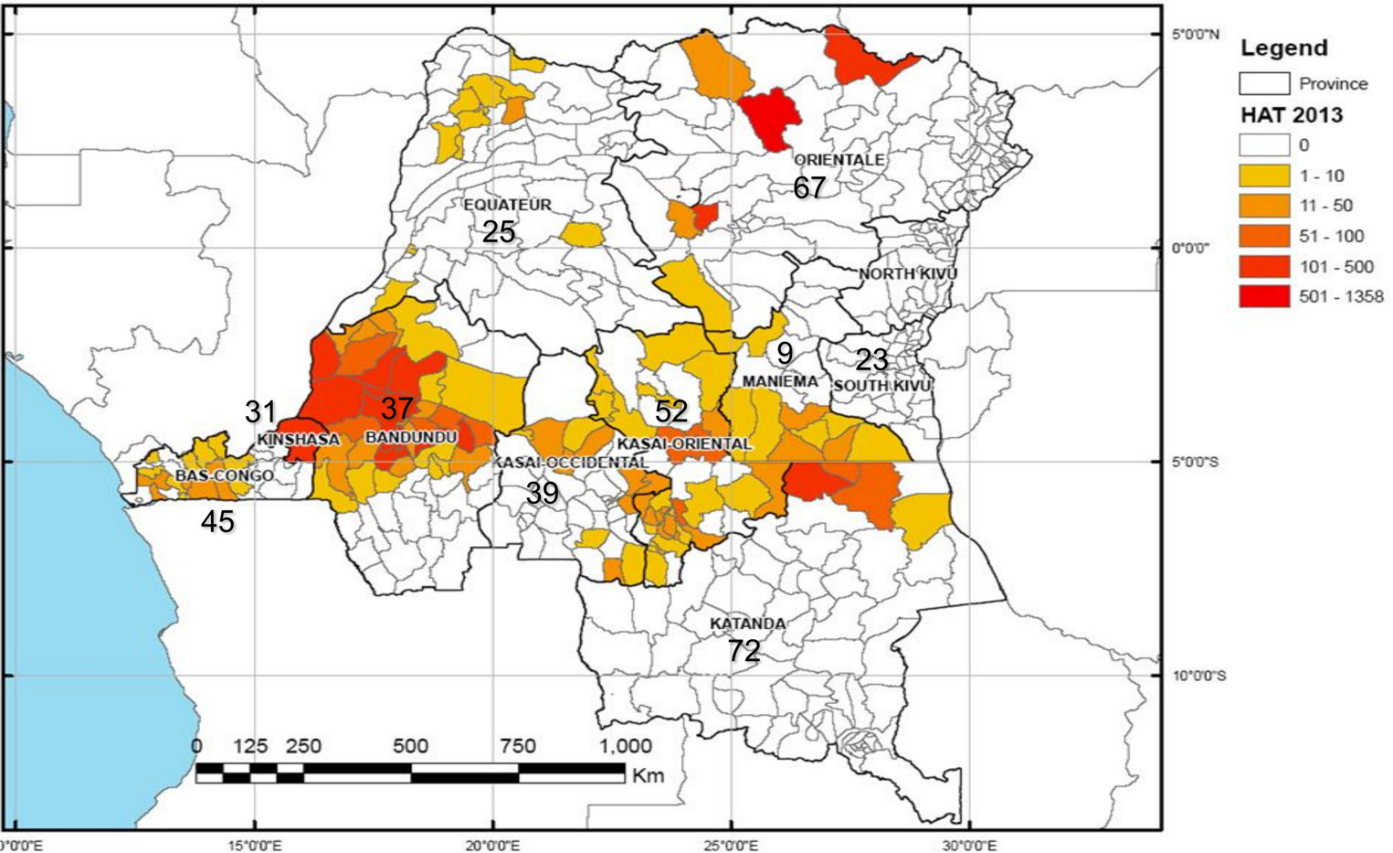
Geographical distribution of participants of the EQA versus HAT incidence. The map represents incidence of human African trypanosomiasis by health zone in the Democratic Republic of the Congo in 2013 [19]. Data represent numbers of health facilities per province.

A total of 445 out of 450 targeted participants were reached during site-visits and 400 answers were received, resulting in a response rate of 89.9% (400/445), 92.0% (368/400) of participants provided answers to all five slides. Fig 1 presents the geographical distribution of EQA participants according to the HAT incidence in DRC in 2013. Referral hospitals and health centers were the most represented facilities (each accounting for more than 40% of all participants) and the distribution was similar for all provinces. Based on PNLP data, the nationwide coverage rates were 39.5% (158/413) and 2.7% (224/8286) for the referral hospitals and health centers respectively [18]. Total cost of the present EQA was approximately 10,000 US$ (salaries of principal investigators not included), with sample preparation and distribution accounting for one third and two thirds respectively.

### Overall performances for the EQA samples

Table 1 presents results for each EQA sample. For slide 1, just over two-thirds of participants (68.6%) reported correctly the absence of parasites. “Malaria” (120/392, 30.6% of participants, mainly *P*. *falciparum)* was the most frequent incorrect answer.

For slide 2, a minority of participants (11.0%) correctly reported the presence of *P*. *malariae*. Over two-thirds (272/383, 71.0%) scored “acceptable”, reporting the diagnosis of malaria, mostly *P*. *falciparum*. The remaining 69 (18.0%) of participants answered “negative” (*i*.*e*. no parasites observed), which was categorised as incorrect.

**Fig 2:**
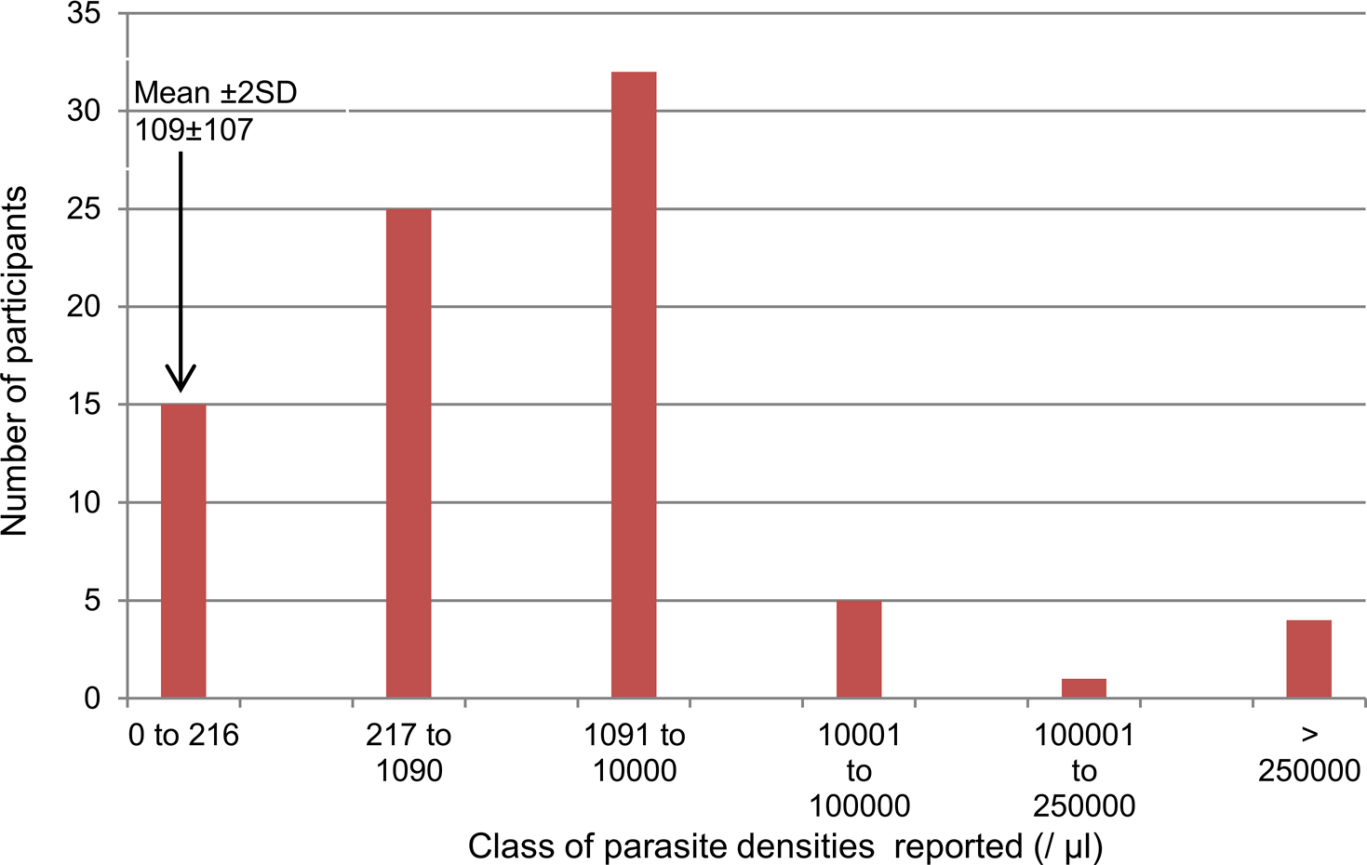
Distribution of parasite densities expressed per μl of blood (n = 82) for slide 3. The bar “0–216” represents parasite densities within 2 SD, the bar “207–1090” represents parasite densities within 10 x the mean value and the rest represent the densities > 10 x the mean value.

For slide 3 (*P*. *falciparum*/*Trypanosoma* spp.), breakdown was as follows: overall (correct, acceptable and incorrect answers combined), 63 (16.5%) of participants reported the presence of *Trypanosoma* and 217 (56.8%) reported malaria: 124 (32.5%) answered *P*. *falciparum* and 93 (24.3%) *Plasmodium* spp. or “positive”. A single participant (0.3%) correctly answered slide 3, reporting the presence of *Trypanosoma* as well as of *P*. *falciparum* at a density of 127/μl, which is within 2 SD of the reference value. Another 23 (6.0%) answers were considered as acceptable, *i*.*e*. answers reporting the presence of both *Trypanosoma* and *Plasmodium* spp. but with a parasite density differing more than 2 SD from the reference value or no parasite density reported at all. The majority of answers (358/382, 93.7%) were incorrect, they included the diagnosis of either only malaria or trypanosomiasis, or the answer “no parasites observed (negative)” (respectively 49.7%, 9.4% and 32.5% of all answers); in addition three participants reported *Trypanosoma* but misdiagnosed *P*. *falciparum* as non-*falciparum*. Among those reporting *P*. *falciparum*, there were 82 (21.5% of all participants) who reported the parasite density (Fig 2): densities were skewed to high values (median of 1100, interquartile (IQR): 262–3377) and in 42 (51.2%) of them, parasite density exceeded 10 times the reference value.

For slide 4 (*Trypanosoma*), less than half (43.4%, 171/394) participants correctly reported the presence of *Trypanosoma*; another 6 (1.5%) answered *Trypanosoma* together with *Plasmodium* spp., which was considered as acceptable. The remaining 217 answers (55.1%) were considered incorrect: they included “no parasites observed” (133/394, 33.8% of participants) as well as malaria (79/394, 20.1% of participants). In slide 4, significantly more participants discovered *Trypanosoma* (177/394, 44.9%) than in slide 3 (63/382, 16.5%, *p* < 0.001) despite lower trypanosome densities in slide 4.

Of note, only 12.9% (49/379) of participants reported the presence of *Trypanosoma* in both slides 3 and 4.

The results for slide 5 (*P*. ovale) were similar to those for slide 2: a minority (24/388, 6.2%) of participants correctly reported the presence of *P*. *ovale;* whereas the vast majority (267/388, 68.8% reported the diagnosis of malaria which was considered as “acceptable”. Nearly a quarter (95/388, 24.5%) answered “negative”, which was considered as incorrect. Only 2.1% (8/381) participants correctly reported the presence of both *P*. *ovale* and *P*. *malariae* on slides 2 and 5 respectively.

Overall, 97.8%, 83.2%, 60.1%, 28.8% and 4.1% of participants (n = 368) reported correct or acceptable answers respectively for at least one, two, three, four and five slides.

### Results of quality of thick blood film routinely prepared and stained by the participants

A total of 280 thick blood film slides (from 70.0%, 280/400 participants) were received for assessment of quality of preparation and staining. One slide was a blank slide with no blood film and was excluded. Table 2 presents the results for the 279 slides retained for assessment: between 41.6% and 76.7% of slides complied with each of the six main quality criteria of staining quality, but only 38 (13.6%) of participants achieved all criteria combined.

**Table 2:** Results for the quality of 279 thick blood film slides prepared and stained by the participants.

Criteria	Numbers (%)
Correct dimensions (> 1 cm) and thickness of the film	214 (76.7)
Thick blood film > 90% intact	155 (55.6)
Complete haemolysis of the red blood cells	134 (48.0)
Chromatin of lymphocytes stained purple	133 (47.7)
White blood cells well distributed on thick blood film	208 (74.6)
No Giemsa stain precipitates observed	116 (41.6)
Complies with all criteria mentioned above	38 (13.6)

### Questionnaire about malaria and HAT diagnosis

Questionnaires were returned by 98.5% (394/400) of participants.

For malaria diagnosis, 99.5% (392/394) participants reported to use thick blood film microscopy; only 0.5% (2/394) of laboratories used exclusively RDTs and 62.4% (246/394) used both thick blood film microscopy and RDTs. A median of two staff members (IQR: 1.0–3.5) per health facility performed a median of 682 (IQR: 300–1,580) thick blood films per semester with a 41.9% (IQR: 14.2–65.0%) positivity rate for malaria diagnosis. The Giemsa stain was used by almost all participants (391/392, 99.7%) and was provided through the PNLP circuit to 32.5% (127/391) participants. The remaining participant used eosin methylene blue. Buffered water was used by 49.3% (175/355) participants, only 11.4% (20/175) of them reported a correct procedure for preparation.

A total of 86.3% (345/400) of participants replied to the questions about HAT diagnosis. A total of 146/345 (42.3%) of them declared not to perform the diagnosis of HAT. For those performing HAT diagnosis, most (94.0%, 187/199) declared to use thick blood film microscopy for HAT diagnosis and nearly one third 32.7% (65/199) reported to use in addition a serological screening test. Serological screening tests mainly included the Card Agglutination Test for Trypanosomiasis “CATT” (used by 90.8% participants (59/65)); the newer rapid sero-diagnostic tests were used by 13.9% (9/65) of them. Use of serological tests was performed by 37/90 (41.1%) referral hospitals (including one provincial laboratory) as well as by 26/98 (26.5%) health centers which had replied to the questions about HAT diagnosis. Concentration techniques such as capillary tube centrifugation (CTC) and mini-anion exchange centrifugation (mAECT) were applied by 15.1% (30/199) and 6.0% (12/199) of participants respectively, the majority of which were referral hospitals (23/32, 71.9%, nine facilities performed both CTC and mAECT). Health facilities performing serological tests and concentration techniques were mainly located in endemic provinces (93.8% (61/65) and 87.5% (28/32) respectively).

A total of 40.2% (157/391) laboratories reported to have received training, but few (15.0%, 59/391) had benefited training focused on malaria and/or HAT during the previous two years.

Among participants who had participated in the previous EQA (136/385), only 48.5% (66/136) had received feedback.

### Factors associated with EQA results

For the 368 participants reporting an answer for all provided slides, Table 3 lists the results for each slide as well as the numbers of participants having at least 4/5 correct or acceptable answers according to health hierarchy. When the group of “other health facilities” (which was composed of private and teaching laboratories) was subtracted, there were no striking differences between the different levels of health facilities. Likewise, there were only small differences among provinces (data not shown).

**Table 3:** Participants reporting and correct answers according to the health care hierarchy, participating in recent training and in previous EQA.

	Nr of participants	Percentage of correct and acceptable answers for each slide					At least 4 answers acceptable
		1	2	3	4	5	
Health care hierarchy							
Provincial laboratories	4	50.0	50.0	25.0	50.0	75.0	25.0
Referral Hospital	149	71.8	81.9	8.7	50.3	69.1	32.2
Referral Health Center	40	65.0	77.5	7.5	47.5	75.0	32.5
Health Center	162	69.1	79.6	0.6	35.8	75.9	22.2
Other health facilities	13	53.8	92.3	38.5	76.9	92.3	61.5
Characteristics of participants							
Training* less than 2 years ago	56	73.2	85.7	10.7	66.1	85.7	42.9
Training* more than 2 years ago, not trained, no date or not specified	312	68.3	79.5	5.4	40.7	71.5	26.3
Participation previous EQA	127	78.0	81.1	13.4	56.7	68.5	40.9
First time participants or not specified	241	64.3	80.1	2.5	38.2	76.3	22.4
Training* less than 2 years ago + Participation in the previous EQA	28	85.7	85.7	17.9	82.1	85.7	57.1
Total	368	69.0	80.4	6.3	44.6	73.6	28.8

*: Training on malaria and/or Human African Trypanosomiasis.

A total of 56/368 (15.2%) participants who answered to all five slides had participated in a training on malaria and/or HAT during the previous two years. Considering the criterion of at least 4/5 correct or acceptable answers as a proxy for good slide reading quality, recently trained participants scored significantly better compared to those who were not recently trained (42.9% (24/56) versus 26.3% (82/312) scored at least 4 questions correct, *p* = 0.01). In particular, recently trained participants scored significantly better for slides 4 and 5 (*Trypanosoma* and *P*. *ovale* respectively) compared to those who were not recently trained (66.1% (37/56) versus 40.7% (127/312), and 85.7% (48/56) versus 71.5% (223/312)) participants reporting correct or acceptable answers for slides 4 and 5 respectively, *p-*values of respectively < 0.001 and 0.03).

Participation in the previous EQA (EQA2011) was reported by 34.5% (127/368) of participants. Participants which had participated in EQA2011 (n = 127) presented higher proportions with correct or acceptable answers than first time participants (n = 241) for slide 1 (78.0% versus 64.3%, *p* = 0.007), slide 3 (13.4% versus 2.5%, *p* < 0.001) and slide 4 (56.7% versus 38.2%, *p* = 0.001). Moreover, a higher proportion of EQA experienced participants obtained a score of at least 4/5 of acceptable or correct answers compared to first time participants (40.9%, versus 22.4%, *p* = 0.001). A larger (but still low) proportion of participants mentioning the use of HAT specific diagnostic tests (CATT, rapid sero-diagnostic tests, CTC and mAECT) scored correct or acceptable answers for the *Trypanosoma* containing slide 3 than those who did not (22/76, 29.0% versus 22/123, 17.9%), but difference was not significant. In contrast, there was no difference between these proportions for the *Trypanosoma* containing slide 4. Recognition of *Trypanosoma* was not significantly different between HAT endemic or non-endemic provinces. For slide 3, the proportion of correctly identified *Trypanosoma* were 17.9% (50/280) among HAT endemic and 12.8% (13/102) among HAT non-endemic, *p* = 0.30. For slide 4, values were 47.1% (138/293) and 38.6% (39/101) respectively, *p* = 0.17. However, participants who declared to perform the diagnosis of HAT identified *Trypanosoma* significantly better than those who declared not to perform the diagnosis of HAT (22.9% (44/192) versus 8.6% (12/139), and 22.5% (44/196) versus 8.3% (12/144)) participants correctly identifying *Trypanosoma* in slides 3 and 4 respectively, *p*-values = 0.001).

There was no difference in proportions of correctly stained thick blood films between participants who procured the Giemsa stain through the PNLP circuit versus those who procured elsewhere, nor between those using buffered water or not (*p* ≥ 0.6); likewise there was any difference for the individual staining quality criteria such as complete haemolysis, correct chromatin staining or presence of precipitates (*p* ≥ 0.6). There was no difference in proportions of correctly stained thick blood films between recently trained participants versus those who were not; the same for laboratories who had participated in the previous EQA versus the first time participants.

## Discussion

The present EQA showed poor performance among diagnostic laboratories in DRC for the microscopic diagnosis of non-*falciparum* malaria and HAT. Performance (in particular the detection of *Trypanosoma*) was better among recently trained and EQA-experienced participants.

This was the third nationwide EQA on blood parasites organised in DRC. In comparison with EQA2010 and EQA2011 [12,13], the numbers of participants had increased (from 174 over 277 to 400), and presently 10/11 provinces were included. Moreover, the coverage rate of health facilities had increased, particularly among the referral hospitals (from 17.4% (72/413) in 2010 to 39.5% (158/413) in the present EQA session). Unexpectedly, only 4/11 provincial laboratories had participated in the present EQA. Despite difficulties of communication and transport infrastructure in DRC (4,007 km and 2,794 km of railway and paved roadways respectively for a surface of 2,345,409 km^2^ [20]), the response rate was 89.9%.

Unlike previous sessions [12,13], the present EQA sessions assessed diagnostic performance of the non-*falciparum* species. Clinical disease and health impact of non-*falciparum* species are less severe compared to *P*. *falciparum* and in DRC, *P*. *falciparum* accounts for > 95% of cases [18], by consequence the need to recognize non-*falciparum* species in daily diagnosis might be questioned. However, missing the diagnosis of non-*falciparum* malaria may delay treatment and may divert the clinician to other diagnosis and additional exams. In that way, it is of note that only 2.1% (8/381) participants correctly diagnosed both *P*. *ovale* and *P*. *malariae*. *Plasmodium* species identification has been shown to be a difficulty in both endemic and non-endemic settings. For instance, assessments conducted through malaria microscopy training of public health and research laboratory personnel in Kenya and Ghana reported erroneous identifications of *Plasmodium* species, particularly for the non-*falciparum* species [21]. In non-endemic settings, EQAs sessions equally reported difficulties to distinguish the non-*falciparum* species (missed *P*. *malariae*, *P*. *ovale* and *P*. *vivax* respectively by approximately 5% up to 70% participants [11,22,23]). Most of concern in the present study was however the fact that one fifth of participants (18.0% and 24.5%) reported “negative” for each of the *non-falciparum* slides, despite relative high parasite densities present on reference-stained Giemsa thick and thin blood films.

The diagnosis of HAT was poor. In slide 3 (*P*. *falciparum*/*Trypanosoma*), less than a fifth (16.5%) of participants recognized *Trypanosoma*, despite their relative high density (1–3 per high power field). The co-presence of *P*. *falciparum*—recognised by over half of participants—may have distracted from further search and diagnosis of *Trypanosoma*. Despite its lower density, *Trypanosoma* was reported by significantly more participants in slide 4 (containing *Trypanosoma* only)–although still by less than half (43.4%) of participants. Combined results for both slides showed that diagnosis of *Trypanosoma* was very low (12.9%, 49/379) compared to that reported from EQA2011 (49.6%, 134/270). Unlike the case in EQA2011, there was neither a better performance in provinces endemic for HAT [13], however the participants who declared to perform the diagnosis of HAT identified *Trypanosoma* better than those who did not.

The slide panel further showed interesting observations about *P*. *falciparum* diagnosis. First, for slide 1 (which contained no parasites), nearly a third (32.4%) of participants reported the presence of blood parasites. Diagnosing (or reporting) malaria in parasite-negative specimens is a well-known phenomenon and has been consistently observed in diagnostic settings in sub-Saharan Africa, with false positive rates ranging from 1.4% to 51.0% [5,24–26]. Despite this, the high proportion of blood parasites presently reported was surprising given the particular setting of an EQA session. It further occurred equally frequent among recently trained participants and contrasted with the 19.0% rate observed in EQA2011 [13]. Slide 3 further demonstrated high failure rate for the detection of low-density *P*. *falciparum* trophozoites (109 ± 107/μl). Microscopy may have a limit of detection of 4–20 parasites/μl but under field conditions, it is rather 50–100 parasites/μl [27,28]. Further, the quality of staining is a main factor decreasing sensitivity in remote settings [5] but could be excluded as a cause of missing parasites, as the present slides were stained in a reference setting. Finally, slide 3 also showed poor performance in the counting of parasite density, confirming findings EQA2010 and EQA2011 [12,13]. The overall staining and preparation quality of the slide from the routine diagnosis was low, with slides from only 13.6% of participants meeting all quality criteria, which was lower than recorded in EQA2010 (19.4%) [12]. The present results are similar to those reported in a recent study in Angola (9.1% and 16.3% good quality of thick blood films before and after training respectively) [5].

In contrast to previous EQA2010 and EQA2011, there was no significant relation with health facility hierarchy or exposure and performance, but better performance, in particular for the detection of *Trypanosoma* was related to being recently trained and EQA experienced but although statistically significant, the actual differences were disappointingly small.

As to the malaria diagnostic tools on site, the coverage rate of RDTs had increased (24.7% and 44.3% in EQA2010 and EQA2011 versus 62.4% presently [12,13]). In addition, it was clear that malaria RDTs (more than 6 million distributed in 2013 [6]) were used as an additional tool next to microscopy, with all but 246 participants who used RDTs declaring to use them as next to microscopy. The strategy of using malaria RDTs, as recommended by the PNLP—use of RDTs in circumstances where microscopy is not feasible—seems not scrupulously implemented in the country [6].

For the diagnostic tools available for HAT, it appeared that thick blood film microscopy was the single available technique in most diagnostic laboratories, despite its inherent low sensitivity of 26–35% for diagnosis of *T*.*b*. *gambiense* HAT [29,30]. Together with the low overall performance of HAT microscopy in slides 3 and 4, this is of concern, as in DRC the diagnosis of HAT has been moving from active screening (mobile teams, CATT) to integration in the horizontal health-care system [8,10,31]. Although not designed to assess the exact coverage rate of HAT diagnosis among health facilities, the present EQA revealed that only one third of participants had sero-diagnostic tests available whereas diagnosis of HAT according to the actual national policy needs to be available at the health center level all over the country [31]. About one fifth of laboratories had concentration techniques such as CTC and mAECT in place, these tools are recommended also for post-therapeutic monitoring of HAT [31] and by consequence are intended to be available in referral hospital level–however they were only reported to be used by a minority.

### Limitations and Strengths

The present EQA shares a number of limitations, intrinsic to EQAs but also related to the particular setting. In general, the overall performance assessed through EQA sessions risks to be overestimated because it can be expected that slides submitted as part of EQA slides will be read by the most experienced microscopists and without constraints of urgency and workload. In addition, many health facilities, particularly in the private sector, had not been addressed and, there may have been a bias as participating health facilities where selected by previous participation, accessibility and existing contacts, which could have had an impact on the results. The situation may therefore be worse among health care facilities not included in the EQA. Related to the present EQA, it should be noted that, apart from the staining and preparation assessment of the routinely prepared thick blood film slide, the present EQA did not address the pre-analytic and post-analytic phases. Another limitation was that the PNLP had not yet established a formal network for diffusion of slides. Despite the extended coverage, logistic difficulties in DRC remain the major challenge [6,32], affecting also distribution of correct feedback.

As to the strengths, there was the increased coverage, particularly among the reference hospitals. Likewise, there was a high response rate (as was the case for previous EQAs [12,13] and according to the current and previous experiences, EQAs organised by INRB were perceived by the participants as didactic rather than as punitive assessments. In addition, the EQA proved to be cost-effective, as 400 participants were reached for a total of 10,000 US$, representing 25 US$ per participant. Part of the cost-effectiveness was assured through collaboration between INRB, the national malaria, tuberculosis and HAT control programs and the provincial health divisions, in line with the World Health Organization recommendation for integrating EQA of the microscopically diagnosed diseases [33,34].

The fact that nearly half of participants who had been participating to EQA2011 declared not to have received the EQA feedback, needs reflection on the EQA organization. Likewise, the turnaround times of the present and previous EQA were long (15 and 21 weeks for EQA2011 and the present EQA respectively). Therefore, at present, systems are considered to shorten the turnaround time of the EQA sessions and to assure swifter communication of the feedback report. To that extent, SMS are explored, as well as pictures transmitted by smart phones [17,35]. A more adequate feedback system should positively influence the impact of the EQA on microscopic quality.

## Conclusion

In conclusion, the present EQA showed poor performance of blood parasite microscopy including non-*falciparum* species and *Trypanosoma* among diagnostic laboratories in DRC. It identified recent training and previous EQA participation as factors associated with a better performance, but also revealed that the EQA result feedback system should be improved.

## Supporting Information

S1 File EQA questionnaire sent to the participants. (DOC)

S2 File Raw data used for writing the manuscript. (XLSX)
