# Supplementary material for: Performance of Microscopy for the Diagnosis of Malaria and Human African Trypanosomiasis by Diagnostic Laboratories in the Democratic Republic of the Congo: Results of a Nation-Wide External Quality Assessment
Source: PLoS One. 2016 Jan 20;11(1):e0146450. doi: 10.1371/journal.pone.0146450 (PMC4720473; doi:10.1371/journal.pone.0146450)
Supplement: S1 File — (DOC) [file pone.0146450.s001.doc]

**Questionnaire d’enquête sur la réalisation du diagnostic microscopique des parasitémies** (A remplir par le chef du laboratoire ou son remplaçant)

Nom de la structure : _________________________________________ Code Labo : _________________

Zone de santé : ________________________________ Province: ________________________________

Type de structure : □Labo provincial □Hôpital (HGR) □Centre de santé de référence

□Centre de santé □Autre (Décrivez) _______________________________________

**Nom du responsable du laboratoire:** ___________________________ Qualification _________________

E-mail : ____________________________________Tél. ________________________________________

1. Quels sont les diagnostics pour lesquels vous utilisez la goutte épaisse ?

Paludisme Trypanosomiase humaine africaine Filariose  Autres (décrivez svp) : ________________________________________

1. Pour le diagnostic du paludisme, vous utilisez : (plusieurs réponses possibles)

les tests de diagnostic rapides (TDRs). Si oui, **Marque du TDR** : _____________________________________

la goutte épaisse

1. Pour le diagnostic de la maladie du sommeil, vous utilisez : (plusieurs réponses possibles)

Nous ne faisons pas le diagnostic de la maladie du sommeil les tests de diagnostic rapides (TDRs) la goutte épaisse le CATT- test la mini-colonne la centrifugation en tube capillaire

1. Combien de gouttes épaisses votre laboratoire a-t-il examinées de janvier à juin 2013? ___________________
2. Combien de gouttes épaisses étaient positives de janvier à juin 2013 pour ? Paludisme ?_________ Trypanosomiase ?_________ Autres (Préciser) ?_________________________
3. Combien des techniciens lisent les GE dans votre laboratoire ? ________________________
4. Avez-vous participé à une formation (recyclage) sur le diagnostic **MICROSCOPIQUE** ? Oui  Non

- Si oui, pour quelle(s) maladie(s) ? __________________________________________________________
- Si oui, la (le) dernière formation (recyclage) remonte à quand ? ___________________________________

1. Avez-vous participé à l’EEQ 19 de 2011 ? Oui  Non.

Si oui, Avez-vous reçu le feedback (réponses correctes) de l’EEQ_19 de 2011 Oui  Non

1. Quel type de colorant utilisez-vous pour la coloration des gouttes épaisses ? ____________________________

Quelle est la marque de votre colorant ? ________________________________________________________

1. Sous quelle forme se présente votre colorant des gouttes épaisses à l’achat ?

Poudre Solution mère Solution de travail Autre (Précisez) _________________________________________________________________________

1. Quelle est la source d’approvisionnement de vos colorants pour la malaria ?

Programme national ONG Fond mondial Centrale d'achat Distributeur local Fournisseur ambulant Autre (Précisez) _________________________

1. Quel type de liquide utilisez-vous pour diluer le colorant  des gouttes épaisses et comment le préparez-vous ? __________________________________________________________________________________________

Fait à _______________________le _________________

Nom et Signature : ________________________________

**Veuillez retourner vos résultats avant le 20 Novembre 2013 !**

**à Mr Pierre Mukadi Kaningu, C/0: INRB, Avenue de la Démocratie, C/ Gombe, B.P. : 1197 Kinshasa I, E-mail :** [**kepha4@hotmail.com**](mailto:kepha4@hotmail.com)**, Tél. : 0898945630 ou 0821444532**
